# Simultaneous detection of *Fusarium culmorum *and *F. graminearum *in plant material by duplex PCR with melting curve analysis

**DOI:** 10.1186/1471-2180-6-4

**Published:** 2006-01-23

**Authors:** Christoph Brandfass, Petr Karlovsky

**Affiliations:** 1 University of Göttingen, Institute of Plant Pathology, Grisebachstraβe 6, 37077 Göttingen, Germany

## Abstract

**Background:**

Fusarium head blight (FHB) is a disease of cereal crops, which has a severe impact on wheat and barley production worldwide. Apart from reducing the yield and impairing grain quality, FHB leads to contamination of grain with toxic secondary metabolites (mycotoxins), which pose a health risk to humans and livestock. The *Fusarium *species primarily involved in FHB are *F. graminearum *and *F. culmorum*. A key prerequisite for a reduction in the incidence of FHB is an understanding of its epidemiology.

**Results:**

We describe a duplex-PCR-based method for the simultaneous detection of *F. culmorum *and *F. graminearum *in plant material. Species-specific PCR products are identified by melting curve analysis performed in a real-time thermocycler in the presence of the fluorescent dye SYBR Green I. In contrast to multiplex real-time PCR assays, the method does not use doubly labeled hybridization probes.

**Conclusion:**

PCR with product differentiation by melting curve analysis offers a cost-effective means of qualitative analysis for the presence of *F. culmorum *and *F. graminearum *in plant material. This method is particularly suitable for epidemiological studies involving a large number of samples.

## Background

Fusarium head blight (FHB) is a disease of cereal crops, which has a severe impact on wheat and barley production worldwide. The infection of heads of small grain cereals and maize plants with *Fusarium *spp. impairs grain yield and quality [1]. Apart from adversely effecting grain size, weight, protein content, baking quality of the flour, and other technological parameters, the contamination of grain and cereal products with *Fusarium *mycotoxins is the most serious consequence of FHB [2,3]. The consumption of commodities contaminated with mycotoxins poses a health risk to both humans and farm animals, making fungal contamination a key concern in food and feed safety assessments. Because grains of low quality are used in feedstuff production rather than in human foods, health and productivity impairment in farm animals caused by mycotoxin contamination of feeds have regularly been reported in the last decades from Europe [4]. Our understanding of the effects of low doses of *Fusarium *mycotoxins consumed over prolonged time periods (possibly the whole life span) on humans is poor because of technical difficulties in addressing these issues by doing epidemiological studies. While the EU is still in the process of developing legal limits for *Fusarium *mycotoxins in grains, food and feeds, some European countries have already established national limits [5,6].

In spite of breeding efforts aiming at FHB-resistant cultivars, and despite a recent shift of the priority of chemical protection development towards fungicides targeting FHB, the disease continues to pose a major challenge to grain growers all over the world [7]. A key prerequisite for a reduction in the incidence of FHB through crop management is an understanding of its epidemiology. The *Fusarium *species primarily involved in FHB are *F. graminearum *and *F. culmorum *[8]. The biology and infection mode of these two species are very different from each other: *F. graminearum *reaches cereal heads via ascospores forcibly discharged from asci formed on plant residues on the soil surface. In comparison, *F. culmorum *does not possess a sexual cycle. It is assumed that *F. culmorum *reaches the ears by traversing from one leave to the next in rain splashes [9], but some researchers find this hypothesis unsatisfactory. A speculation that *F. culmorum *and possibly also *F. graminearum *can infect cereal plants systemically and grow in the stem upwards from the root to the ear has been revived repeatedly in recent years, but it has not been proven so far [10].

Another important question in FHB epidemiology is whether species other than from *F. graminearum *and *F. culmorum *also contribute significantly to the symptoms and mycotoxin contamination. A number of fungal species have been isolated from infected or even healthy-looking ears collected in the field, including a plethora of *Fusarium *spp. [11,12], but it is not known whether these isolates actively contribute to the FHB or whether they just grow saprophytically in the dead surface tissues, which are normally removed during grain cleaning and processing.

Current epidemiological studies on FHB are expected to provide answers to these and other questions crucial to disease management. These studies require the analysis of a large number of samples in a species-specific manner. Although the quantitative determination of fungal biomass is required for certain tasks (e.g., virulence assessment for species or isolates), qualitative data are often sufficient in studies in epidemiology. PCR is the method of choice for both purposes, because reliable species-specific primers are known for all FHB-relevant species [12-17]. However, classical PCR is not suitable for epidemiological studies because agarose electrophoresis limits the throughput [18,19]. Here we describe a fast, inexpensive, qualitative duplex PCR assay for *F. culmorum *and *F. graminearum*, which are the key species causing FHB in wheat and barley. The method is based on the melting curve analysis of amplification products generated in a real-time PCR thermocycler.

## Results and discussion

### Optimization of DNA extraction and PCR conditions

The processing of the wheat rachides posed a challenge, because the small sample size (2–3 segments of the spike axis per sample) and firm texture of the material put high demands on the homogenization of the samples. The high number of samples precluded manual grinding in a mortar; we therefore used a reciprocal mill. Pulverizing wheat rachides in polypropylene tubes with stainless steel or wolfram carbide balls was found to be inefficient, but a large stainless steel ball (32 g, 20 mm) in a stainless steel container crushed the rachides into a fine powder. We optimized the factors affecting the DNA yield and quality in a CTAB-based DNA extraction method (details to be published), the optimized protocol is described in the Methods section.

Our initial PCR experiments, undertaken under the conditions described in the literature for classical PCR with primers Fg16N and OPT18, did not result in the amplification and detection of the expected products by fluorescence [13,14]. Therefore, we optimized the PCR conditions concerning primer concentration, polymerase activity, MgCl_2_, SYBR Green I concentration and cycling parameters. For this optimization fungal DNA of the strains Fg3, Fg5, Fc2 and CBS 250.52 (Table 1) was used. The improved protocol is described in the Methods section.

The specificity of primers we selected for our assay was evaluated in the literature as follows: Fg16N was tested with 21 *F. culmorum *isolates, 24 *F. graminearum *isolates, 20 isolates of other *Fusarium *species and 5 isolates of other fungal species associated with cereals [14]. OPT18 was tested with 69 *F. culmorum *isolates, 34 *F. graminearum *isolates, 25 isolates of other *Fusarium *species and 27 isolates of other fungal species associated with cereals [13]. All tests confirmed the specificity of Fg16N for *F. graminearum *and OPT18 for *F. culmorum*. Because PCR conditions used in these tests were different from the conditions of our assay, we re-evaluated the primer specificity in our duplex assay. We tested a range of fungal species regularly encountered on cereals, especially *Fusarium *spp. (Table 1). All isolates of *F. culmorum *and *F. graminearum *tested positively, giving signals in the expected range of melting temperature. There was no cross-reaction between *F. culmorum *and *F. graminearum*. All other fungal isolates listed in Tab. 1 tested negatively. The amplification of *F. culmorum *and *F. graminearum *was not inhibited by the presence of a large excess of wheat DNA (10 ng wheat DNA in a reaction containing 10 pg of *Fusarium *spp. DNA) nor did pure wheat DNA generate any signal under these conditions.

### Duplex PCR with SYBR Green I and melting curve analysis

**Figure 1:**
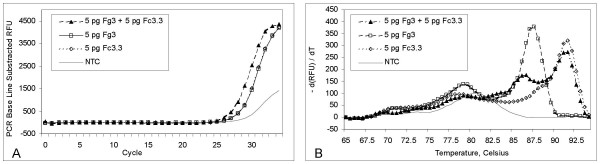
**Progress and melting curves of PCR products used for the detection of *F. culmorum *and *F. graminearum***. PCR and melting curve analysis was performed in a real-time thermocycler with primer pairs specific for *F. culmorum *and *F. graminearum *and SYBR Green I fluorescence detection as described in the Methods. The SYBR Green I was diluted to 0.4× the recommended concentration. Genomic DNA from *F. graminearum *(continuous curves with squares), *F. culmorum *(dotted curves with diamonds), a mixture containing DNA from both species (dashed curves with triangles) and no DNA (no-template control, "NTC ", gray curve without symbols) were used as templates. A: Progress curves, showing the fluorescence signal during the annealing phase in each reaction cycle. B: Melting curves recorded on PCR products after 35 cycles. The rise of fluorescence in the no-template control produces a melting point maximum at about 80°C, far lower than where *Fusarium *target are detected. Because melting curves presented in Figure 1B were recorded with low template amounts, the unspecific products are also visible, but they do not prevent the detection of *Fusarium *DNA in amounts equal to or higher than the limit of detection.

The fluorescent dye SYBR Green I, which intercalates unspecifically to all double-stranded DNA products, is commonly used in real-time PCR when just one specific amplicon is produced. In our protocol, the dye serves to identify two different products after amplification with the help of a melting curve analysis. In contrast to the real-time PCR mode, this method does not allow for quantification because the fluorescence measured during amplification is the sum of the signals generated by two specific PCR products and possibly unspecific products, too. Particularly, the primer pair Fg16N F/R tends to generate a fluorescent signal even in no-template controls at the end of the amplification (Figure 1).

A melting curve analysis was set up to characterize the amplification products after PCR. Melting points, represented by maxima of the first derivative of the melting curves, were different for the two PCR products in our system. A low heating rate (0.5°C 30 s^-1^) during melting curve acquisition was essential because the resolution of the melting curves drops with increasing heating rate.

To reduce the fraction of false positives, absolute and relative limits were set for the magnitude of signals represented by peak areas on the first derivative of the melting curves. The absolute peak area and the fraction of the peak area in relation to the total area under the melting curve were used as the absolute and relative thresholds. Their limits were respectively set to 100 and 0.1.

The melting temperature of a PCR product depends mainly on its length and GC content. It is also affected by the nucleotide sequence because of the stacking interactions among adjacent nucleotides. The species-specific PCR product for *F. culmorum *generated with primers OPT18 F/R was 472 bp long and had a melting temperature of 91.0°C ± 1°C in the PCR buffer, whereas the product for *F. graminearum *was 280 bp long and had a melting point of 86.5°C ± 1°C (Figure 1). A third, wide-shaped peak occasionally occurred between 76 and 82°C. This originated from "primer dimers" and other unspecific amplification products. These latter products were found particularly in samples with a low concentration of *Fusarium *DNA. As the result presented in the Figure 1 shows, unspecific products do not prevent the detection of *Fusarium *DNA in amounts equal to or higher than the limit of detection (Table 2). The nonspecific products in the no-template control possess a melting point of about 80°C, which is much lower than the melting temperatures at which *Fusarium *targets are detected.

Experiments with primer pairs used separately revealed that the unspecific products were produced by primers Fg16N F/R, while primers OPT18 F/R did not generate any unspecific products even in controls with no template DNA.

In contrast to the commonly held view that SYBR Green I binds equally well to all double-stranded DNA sequences, we observed that the *F. culmorum *product binds the dye more efficiently than the *F. graminearum *product. Similar differences in the binding affinity of SYBR Green I to different PCR products have been reported by Giglio et al. [20]. Because of this preferential binding of the dye to the *F. culmorum *product, *F. graminearum *could not be detected by melting curve analysis in the presence of 100 pg or more of *F. culmorum *DNA, irrespective of the amount of *F. graminearum *DNA. Attempts to counteract this effect by increasing the SYBR Green I concentration in the PCR reaction mixture were hampered by the inhibition of the PCR by the dye. Since even the addition of more SYBR Green I after the completion of the PCR did not prevent the suppression of the *F. graminearum *signal by the *F. culmorum *DNA (data not shown), we consequently re-optimized the PCR conditions for *F. graminearum *detection in the presence of 5 and 100 pg *F. culmorum *DNA (see Table 2).

During the re-optimization of the PCR conditions we noticed that increasing the SYBR Green I concentration caused an increase in the melting temperature of the PCR products. Table 3 shows the results of a systematic investigation of this phenomenon for the *F. graminearum *product. A shift caused by increasing the SYBR Green I concentration from 0.1× to 0.7× dilution amounted to as much as 2°C.

The detection limits of the melting curve analysis are summarized in Table 2. The assay is used for samples with a low amount of total DNA (e.g., DNA extracted from rachides of cereal ears) or when merely one product was expected (e.g. identification of pure cultures or *Fusarium *spp. isolates). Therefore, we recommend keeping the total amount of *Fusarium *spp. DNA in the assay under 50 pg when the presence of both species is expected. This can be achieved by employing amounts smaller than 1 ng plant DNA in the reaction or by monitoring the C_T _value which has to be bigger than 20.

Because the suppression of the *F. graminearum *signal by *F. culmorum *only occurs when the PCR products are characterized by the melting curve analysis, agarose gels can be used to confirm the interpretation of the melting curves when large amounts of *F. culmorum *DNA are detected. This is characterized by a signal (melting curve maximum) specific for *F. culmorum *and a C_T_-values of the whole amplicon smaller than 25. In our study of 500 wheat samples, no *F. graminearum *signal was inhibited by *F. culmorum *DNA (see below).

### Data processing

The form in which the results of the melting curve analysis are provided by real-time thermocyclers has not been designed for automated evaluation or a high-throughput environment. To facilitate the interpretation of the results of a multiplex melting-curve-based PCR analysis, we designed a spreadsheet which transformed the output from the thermocycler into a user-friendly, color-enhanced tabular report. In addition to the presentation of the results, the spreadsheet integrates a convenient plate setup (arrangement of samples in the PCR plate) with a pipetting scheme for the reaction mixtures. The data processing and reporting tool consists of eight spreadsheets in Microsoft Excel 2000 (see Additional file 1).

The first sheet "Plate Setup" can be customized according to the user's needs. The content of the plate setup sheet, including identifiers, is used by the interpretation sheet "Data Analysis". The second sheet "Pipetting Scheme" just helps to calculate pipetting, it does not contain any link to the remaining parts of the file. Raw data from the iCycler are copied into the third and fourth sheets: the C_T _table ("PCR Quantification" menu) is copied into the third Excel sheet called "CT Table" and the Peak table ("Melt curve" menu) is transferred into the fourth Excel sheet designated "Peak Table". These two sheets, together with the identifiers taken from the plate setup, are the basis for the "Data Analysis" sheet which presents the final results. The auxiliary sheet "Adaptation" contains merely some formatting schemes used by the "Data Analysis" sheet, it does not need to be dealt with by the user. The sixth sheet "Print Sheet" is an empty sheet which can be used for print format adjustments by the user after data from the "Data Analysis" sheet has been copied on to it, because editing options are limited within the "Data Analysis" sheet. The final sheet "Manual" consists of a manual describing the use of the spreadsheet. The spreadsheet file can be downloaded from [21].

**Figure 2:**
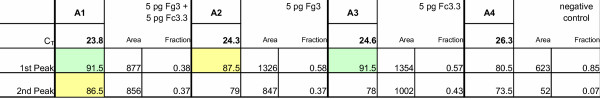
**Section of the interpretation sheet corresponding to 4 wells**. Each result consists of a well position, sample identifier, C_T _value, melting temperatures, areas under the melting curve peaks, and fractions of these areas in relation to the total area under the melting curve. The melting data are calculated for the two products with the highest melting temperatures only. Fields are automatically highlighted in a species-specific manner (green: *F. culmorum*, yellow: *F. graminearum*) and a warning is issued by a red coloring of the fields when the peak size does not reach the preset absolute and relative minimal values. In addition, if there is an unexpected third melting-curve maximum at temperatures higher than 80°C (indicating the presence of additional PCR products from *F. culmorum *or *F. graminearum*), the corresponding well will be highlighted in orange to indicate that the user should check the raw data.

The information given for each well in the PCR plate consists of a well identifier, C_T _value, melting temperatures, areas under the peaks in the first derivative of the melting curve, and the fraction of the peak area in relation to the total area under the curve (Figure 2). Only these two peaks, corresponding to the products with the highest melting temperatures, are processed. The calculation of the areas under the peaks in the first derivative melting curves is an undocumented feature of the iCycler software: these values are not available within iCycler interface, but they are transferred together with other melting curve data when the table is copied to a spreadsheet via the clipboard. To work with other thermocyclers, the spreadsheet presumably will need to be modified according to the format used to export the melting curves.

The interpretation of the results is facilitated by an automatic highlighting of the important fields: the presence of *F. culmorum *(melting temperature between 90.0 and 92.0°C) is indicated by green and *F. graminearum *(melting temperature between 85.5 and 87.5°C) is colored yellow. If the peak area or the fraction of the peak area in relation to the total area under the melting curve does not reach the set quality limits (see previous section), the fields will be colored red to warn of potential false positives. These limits can be adjusted centrally in the head of the interpretation sheet, based on the intensity of the signals produced by the DNA standards.

In some cases, there are more than two melting curve maxima at temperatures higher than 80°C where the PCR products of *F. culmorum *and *F. graminearum *are to be expected. In such a rare case, the corresponding well will be marked orange in the interpretation sheet. This is an indication that the raw data should be checked.

### Analysis of field samples

The method was tested by analyzing 500 DNA samples extracted from the rachides of wheat heads with symptoms of FHB sampled throughout Germany in 2003. At least one of the two fungi was identified in all but 21 samples. Both pathogens were detected in 14 rachides. In 398 rachides, only *F. graminearum *could be detected and 67 samples showed only a *F. culmorum *signal. The latter samples were additionally analyzed for the presence of *F. graminearum *by PCR, in which the *F. culmorum*-specific primers were omitted to see whether the *F. graminearum *signals would be suppressed by *F. culmorum *DNA. This single-target analysis confirmed the results of duplex PCR for all 67 samples, demonstrating that the suppression of the *F. graminearum *signals by *F. culmorum *DNA did not occur in any of the 500 samples analyzed.

Therefore, we suggest that a confirmatory analysis (by a single-target PCR or by gel electrophoresis of products of duplex PCR analysis) for samples positive for *F. culmorum *but not *F. graminearum *is unnecessary when working with wheat rachides. However, an extra effort for a confirmatory analysis is rather low (13% of samples in our case). The application of a duplex assay leads to a significant reduction of the sample number as compared to using two separate assays, even when samples positive for *F. culmorum *and negative for *F. graminearum *are analyzed a second time for confirmation.

A standard method for the taxonomic characterization of field samples regarding their colonization with *Fusarium *spp. consists of the isolation of pure cultures on PDA plates, growing the isolates for 7 days on SNA plates or carnation leaf agar and the examination of spores in a microscope. An additional taxonomically relevant marker is the color of fungal colonies on PDA plates. We performed this analysis on 10 rachis samples from ears with FHB symptoms. All samples contained *F. graminearum*, which was confirmed by melting curve analysis-based PCR. In order to verify the PCR method for *F. culmorum*, we selected from a larger set 10 field samples previously identified as containing *F. culmorum *by morphological characters and subjected these samples to melting curve analysis-based PCR. All ten samples tested positive for *F. culmorum*.

To compare both methods, morphological taxonomy requires cultivation for at least 12 days followed by time-consuming microscopic examination. In contrast, the PCR method delivers the results within two days and facilitates parallel processing.

## Conclusion

The method developed in this study allows the simultaneous identification of *F. graminearum *and *F. culmorum *in one PCR without the use of doubly labeled hybridization probes or electrophoresis. Species-specific PCR products are differentiated by melting curve analysis. The detection limit for each single species was 5 pg of genomic DNA. An excess of one species impairs the detection limit for the other species due to the competition of the PCR products for binding of the fluorescent dye used for detection. Because *F. culmorum *products bind SYBR Green I better than *F. graminearum *products, large amount of *F. culmorum *DNA impairs the detection of *F. graminearum*. This effect was partially compensated for by increasing the dye concentration. This competition for dye binding does not play a significant role as long as the amount of *F. culmorum *DNA is low. The assay described in this study is suitable for fast and cost-effective analysis of a large number of samples for the presence of *F. culmorum *and *F. graminearum*. It will be particularly useful in epidemiological studies, as exemplified by the investigation of a large collection of rachides isolated from wheat ears exhibiting symptoms of FHB.

## Methods

### Fungal cultures

The fungal strains used are listed in Table 1. The fungal cultures were maintained at 10°C on SNA medium (0.5 g l^-1 ^MgSO_4 _× 7 H_2_O, 1 g l^-1 ^KNO_3 _(Roth, Karlsruhe, Germany), 0.2 g l^-1 ^sucrose, 0.2 g l^-1 ^glucose, 0.5 g l^-1 ^KCl, 1 g l^-1 ^KH_2_PO_4 _and 15 g l^-1 ^agar (Merck, Darmstadt, Germany)). The *Fusarium *spp. cultures for DNA extraction were grown for 10 days in 100 ml Potato Dextrose Broth (PDB, 24 g l^-1^; Scharlau, Barcelona, Spain). The fungal mycelium was harvested by filtration and freeze-dried.

### DNA isolation from pure fungal cultures grown in liquid media

We used a variant of the CTAB method [22], simplified by others [e.g. [23]], and modified in our laboratory as follows. The lyophilized mycelium (200 mg) was pulverized in a mortar with a small amount of sand (Riedel-de Haen, Hanover, Germany). The ground mycelium was transferred into a 50-ml centrifugation tube containing 10 ml of TES buffer (100 mM Tris (Roth, Karlsruhe, Germany), 20 mM EDTA (Merck, Darmstadt, Germany), 1% (w/v) SDS (Biomol, Hamburg, Germany), pH set to 8.0 with HCL) and 4 mg proteinase K (Merck, Darmstadt, Germany). The lysis mixture was incubated at 45°C for 45 min and the content of the tubes was mixed thoroughly by turning the tubes every 10 min. Subsequently, 3.9 ml of 5 M NaCl (Fluka, Buchs, Switzerland) were added and the sample was mixed before adding 1.4 ml of 10% (w/v) cetyltrimethylammonium bromide (CTAB, Merck, Darmstadt, Germany). The samples were incubated for 10 min at 65°C, cooled in an ice/water bath, and then 10 ml chloroform-isoamyl alcohol (24:1, Roth, Karlsruhe, Germany) were added. After mixing the emulsion thoroughly, the tubes were kept over night in an ice/water-bath. The upper phase (including a small part of the lower phase) was transferred into another centrifugation tube by pipetting and spun for 20 min at 4,000 g (5°C). The watery phase was transferred to a new tube containing 10 ml isopropanol (Roth, Karlsruhe, Germany) at room temperature, mixed thoroughly and centrifuged for 10 min at 4,000 g and room temperature. The supernatant was decanted and the pellet was rinsed with 70% (v/v) ethanol (Roth, Karlsruhe, Germany), dried and dissolved in 4.5 ml TE (10 mM Tris, 1 mM EDTA, pH set to 8.0 with HCl). In spite of the large volume of buffer used to dissolve the DNA, the process took 6 h or longer in some extractions. Dissolving the pellet can be speeded up by heating the tubes to 40°C and then mechanically destroying the pellet. Undissolved material was removed by centrifugation; the DNA was concentrated by ethanol precipitation (1/10 vol. of 5 M ammonium acetate (Merck, Darmstadt, Germany) and 2.5 vol. of 96% (v/v) ethanol) and dissolved in 0.5 ml TE. The quality and quantity of DNA were assessed by electrophoresis in 0.8% (w/v) agarose gels (Cambrex, Rockland, ME, USA) prepared in TAE buffer (40 mM Tris, 1 mM EDTA, pH set to 8.5 with acetic acid, Riedel-de Haen, Hanover, Germany). The electrophoresis was carried out at 4 V cm^-1 ^for 60 min. Double-stranded DNA was stained with ethidium (ethidium bromide, 2 mg l^-1^), (Applichem, Darmstadt, Germany). The gels were documented with the help of a digital imaging system (Vilber Lourmat, Marne la Vallee, France). Densitometry values were compared with those of Lambda Phage DNA (methylated, from *Escherichia coli *host strain W3110, Sigma, Taufkirchen, Germany). The densitometry was performed using Multi Analyst-Software (BioRad, Hercules, CA, USA).

### Isolation of fungi from wheat rachides

To identify the infectious agent causing the FHB, rachides of symptomatic heads were isolated and dissected into pieces spanning 2–3 segments of the spike axis. The rachis samples were rinsed with tap water and surface-sterilized by incubating for 3 min in 3% (v/v) sodium hypochlorite solution (Roth, Karlsruhe, Germany). They were then dried, inserted into agar in PDA-plates (PDB + 1.5% (w/v) agar) and incubated at 20°C for 3–5 days. The mycelia growing out of the rachides were preserved by freezing in 15% (v/v) glycerol (Roth, Karlsruhe, Germany) at -80°C.

### DNA extraction from plant material

The rachides were dried at 60°C over night and a single section (2–3 segments) was ground in a ball mill (Mixer Mill MM 200, Retsch, Haan, Germany) in a stainless steel container with a 20-mm, 32-g steel sphere (Retsch, Haan, Germany) for 30 s at maximum speed. The milling container was rinsed with 1.4 ml CTAB-buffer (10 mM Tris, 20 mM EDTA, 0.02 M CTAB, 0.8 M NaCl, 0.03 M N-laurylsarcosine (Fluka, Buchs, Switzerland), 0.13 M sorbitol, 1% (w/v) polyvinylpolypyrolidone, pH set to 8.0 with NaOH (Merck, Darmstadt, Germany). Two μl mercaptoethanol (Fluka, Buchs, Switzerland) and 20 μg proteinase K (from a stock solution 20 mg/ml) were added shortly before use and the mixture was transferred to a 2-ml tube. After an initial incubation period of 10 min at 42°C and a second incubation for 10 min at 65°C, during which the content of the tubes was mixed each 3 min, 0.8 ml of chloroform-isoamyl alcohol (24:1) were added. The samples were then thoroughly emulsified and centrifuged for 10 min at 5,000 g at room temperature. 750 μl of the upper phase were transferred to a 1.5-ml tube containing 500 μl isopropanol, mixed, incubated for 20 min at room temperature and centrifuged for 15 min at 15,000 g at room temperature. The pellet was washed with 70% (v/v) ethanol, dried and dissolved in 200 μl TE. To ensure that the DNA was dissolved completely, the sediment covered by the TE buffer was incubated over night at 4°C. The quality and concentration of DNA were assessed by agarose electrophoresis as described above.

### DNA extraction from the mycelia grown on agar plates

To extract the DNA from a mycelium growing on the surface of a small agar plaque, the protocol for plant material (see above) was slightly modified. At the beginning of the procedure, the agar plaque was homogenized with 1 ml CTAB-buffer in a reciprocal mill in a 2-ml tube with 9 wolfram carbide spheres (diameter 3 mm, Retsch, Haan, Germany). At the end of the procedure, the DNA was dissolved in 100 μl TE-buffer. A 1:100-dilution was used in the PCR.

### PCR amplification

The iCycler System (BioRad, Hercules, CA, USA) was used for amplification and melting curve analysis. Primers Fg16N F (ACAGATGACAAGATTCAGGCACA) and Fg16N R (TTCTTTGACATCTGTTCAACCCA) were used to amplify a 280 bp fragment specific for *F. graminearum *[14]. Primers OPT18 F (GATGCCAGACCAAGACGAAG) and OPT18 R (GATGCCAGACGCACTAAGAT) served to multiply a 472 bp fragment specific for *F. culmorum *[13]. All the primers were synthesized by Operon Biotechnologies (Cologne, Germany). Both primer pairs Fg16N F/R and OPT18 F/R were derived from randomly amplified genomic fragments, the function of target sequences is unknown.

The amplification mix consisted of 1× NH_4_-reaction buffer (diluted from 10× NH_4_-reaction buffer: 160 mM (NH_4_)_2_SO_4_, 670 mM Tris-HCl, 0.1% (v/v) Tween-20, pH 8.8 at 25°C, Bioline, Luckenwalde, Germany), 5 mM MgCl_2 _(Bioline, Luckenwalde, Germany), 0.2 mM of each dATP, dTTP, dCTP and dGTP (Bioline, Luckenwalde, Germany), 0.3 μM of each primer, 0.7 u BIOTaq DNA polymerase (Bioline, Luckenwalde, Germany), 10 nM Fluorescein (BioRad, Hercules, CA, USA, diluted from 1 μM in 1× NH_4_-reaction buffer, to collect well factors, specific for the iCycler), 0.4× SYBR Green I solution (Invitrogen, Karlsruhe, Germany), 1 μl of template DNA and ddH_2_O up to 25 μl. The detection of amplification products, based on the fluorescence of SYBR Green I, was performed with filters set at 490 ± 10 nm for excitation and 530 ± 15 nm for emission.

The PCR was performed with the following cycling protocol. Initial denaturation for 1.5 min at 95°C (the denaturation time is used by the thermocycler to collect data for the calculation of the well correction factors, which is needed to compensate for differences among wells of the microtitre plate) was followed by 35 cycles with 30 s at 94°C, 45 s at 64°C, and 45 s at 72°C. The final elongation was performed for 5 min at 72°C. During the PCR, the detection of fluorescence was carried out in the annealing step of each cycle. Following amplification, the melting curves were acquired by heating the samples to 95°C for 1 min, cooling to 55°C for one min and then slowly increasing the temperature from 65°C to 95°C at the rate of 0.5°C 30 s^-1^, with a continuous measurement of the fluorescence.

When an electrophoretic analysis of PCR products was necessary, 4 μl of the reaction mixture were combined with 2 μl of loading buffer (100 mM EDTA, 50% (v/v) Glycerol, 0.025% (w/v) bromphenol-blue, Merck, Darmstadt, Germany) and loaded on a 1.7% (w/v) agarose gel prepared in TAE buffer. The electrophoresis and the documentation were carried out as described above.

### Data analysis

Sample identifiers, C_T _values (threshold cycles) and the results of the melting curve analysis were exported from the thermocycler into the pre-formatted Excel spreadsheets, which facilitated the automatic data processing and reporting (see Additional file 1). The Excel file can be downloaded from [21], its structure and function is described in detail in the Data Processing paragraph of the Results section.

## Authors' contributions

CB carried out the experiments, designed the spreadsheets and drafted parts of the manuscript. PK conceived and guided the study, and wrote parts of the manuscript. Both authors read and approved the final version of the manuscript.

## Supplementary Material

Additional File 1 **DuplexPCRFusarium**. The pre-formatted Excel spreadsheet facilitates automatic data processing and reporting. In short, data concerning the two largest maxima identified on the first derivative of each melting curve are processed as follows: the size of the peaks are checked against pre-set minimal values, the melting temperatures are compared with values expected for both species-specific PCR products, and the final results are presented in a tabular report. Click here for file

## Figures and Tables

**Table 1:** Fungal strains used in the present study. Source code: 1: Centraalbureau voor Schimmelcultures, Utrecht, The Netherlands; 2: Deutsche Sammlung von Mikroorganismen und Zellkulturen, Braunschweig, Germany; 3: E. Möller, University of Hohenheim, Germany; 4: H. Nirenberg (BBA Berlin, Germany) via E. Möller, University of Hohenheim, Germany; 5: Institute of Plant Pathology, University of Göttingen, Germany; 6: International Center for Agricultural Research in the Dry Areas, Aleppo, Syria; 7: J.F. Leslie (Kansas State University, Manhattan) via E. Möller, University of Hohenheim, Germany; 8: National Institute for Agricultural Research, Paris, France; 9: Th. Miedaner (State Plant Breeding Institute) via E. Möller, University of Hohenheim, Germany

**Species**	**Strain**	**Source**	**Origin**
*Alternaria alternata*	A 4.1.1	5	*Triticum aestivum*, leaf, 1983
*Cladosporium herbarum*	CH 3	3	unknown
*Cladosporium herbarum*	CH 4	3	unknown
*Drechslera sorokiniana*	D 3.1	5	*Triticum aestivum*, leaf
*Fusarium acuminatum*	ICARDA 92090	6	*Hordeum vulgare*, root, Bouider station
*Fusarium acuminatum*	ICARDA 92099	6	*Hordeum vulgare*, Bouider station
*Fusarium acuminatum*	ICARDA 93682	6	*Hordeum vulgare*, root
*Fusarium acuminatum*	ICARDA 93803	6	*Hordeum vulgare*, root, Viransehir
*Fusarium acuminatum*	ICARDA 93831	6	*Hordeum vulgare*, root, Viransehir
*Fusarium avenaceum*	DSM 62161	2	*Dianthus caryophyllus*, rotten stem base, Germany
*Fusarium avenaceum*	FA 95	3	unknown
*Fusarium compactum*	ICARDA 93823	6	unknown
*Fusarium crookwellense*	BBA 63558, DSM 8704	4	unknown
*Fusarium crookwellense*	BBA 64483	4	unknown
*Fusarium crookwellense*	BBA 64545	4	unknown
*Fusarium culmorum*	CBS 122.73	1	*Triticum *sp., Great Britain, 1973
*Fusarium culmorum*	CBS 250.52 (Fc3.3)	1	*Secale cereale*, grain, 1952
*Fusarium culmorum*	DSM 62184	2	*Zea mays*, grain, 1952
*Fusarium culmorum*	DSM 62191	2	*Triticum aestivum*, head, Mainz, Germany
*Fusarium culmorum*	Fc15	9	*Triticum aestivum*, seed, Söllingen, Germany, 1987
*Fusarium culmorum*	Fc2	4	*Hordeum vulgare*, head, Finland, 1982
*Fusarium culmorum*	Fc22	9	*Secale cereale*, stem, Bad Schönborn, Germany, 1989
*Fusarium culmorum*	Fc36	9	*Secale cereale*, stem, Bergen (Celle), Germany
*Fusarium culmorum*	Fc65	9	*Secale cereale*, Svalöf, Sweden, 1992
*Fusarium culmorum*	Fc66	3	*Triticum aestivum*, head, Italy, 1992
*Fusarium culmorum*	Fc67	3	*Triticum aestivum*, head, Italy, 1992
*Fusarium culmorum*	Fc73	3	soil, Tasmania, Australia
*Fusarium equiseti*	ICARDA 93002	6	unknown
*Fusarium equiseti*	ICARDA 93532	6	unknown
*Fusarium equiseti*	ICARDA 93574	6	unknown
*Fusarium equiseti*	ICARDA 93715	6	unknown
*Fusarium equiseti*	ICARDA 93788	6	unknown
*Fusarium graminearum*	BBA 62048	4	*Triticum aestivum*, Germany, 1973
*Fusarium graminearum*	CBS 389.62	1	*Triticum aestivum*, East-Flevoland, The Netherlands, 1961
*Fusarium graminearum*	DSM 1095	2	*Zea mays*, Indiana, USA, 1973
*Fusarium graminearum*	DSM 4527	2	*Zea mays*, Bavaria, Germany, 1982
*Fusarium graminearum*	DSM 4528	2	*Zea mays*, Germany, 1982
*Fusarium graminearum*	DSM 62217	2	*Zea mays*, Ireland, 1968
*Fusarium graminearum*	DSM 62722	2	*Lolium perenne*, rotting base, Germany
*Fusarium graminearum*	DSM 62722	2	*Lolium *sp., Kiel, Germany, 1968
*Fusarium graminearum*	DSM 64848	2	*Triticum durum*, Rottenburg, Germany, 1987
*Fusarium graminearum*	DSM 67638	2	*Triticum aestivum*, Bavaria, Germany, 1992
*Fusarium graminearum*	Fg3	4	*Triticum durum*, head, 1985
*Fusarium graminearum*	Fg5	4	unknown
*Fusarium oxysporum*	SAGW 124	5	Aalsmeer, The Netherlands, 1987
*Fusarium poae*	DSM 62376	2	*Avena sativa*, kernel, Germany
*Fusarium poae*	FP 2	9	unknown
*Gibberella fujikuroi*	B-00281	7	*Saccharum officinarum*, Taiwan
*Gibberella fujikuroi*	B-01722	7	unknown
*Gibberella fujikuroi*	B-03821	7	unknown
*Gibberella fujikuroi*	B-03828	7	unknown
*Gibberella fujikuroi*	B-03853	7	unknown
*Microdochium nivale *var. *majus*	GN 7	9	*Hordeum vulgare*, head, Switzerland
*Microdochium nivale *var. *nivale*	GN 25	9	*Hordeum vulgare*
*Microdochium nivale *var. *nivale*	GN 35	9	*Secale cereale*, base, Georgen
*Microdochium nivale *var. *nivale*	GN 36	9	*Secale cereale*, base, Oli/Schwäbische Alb, Germany
*Pseudocercosporella herpotrichoides*	INRA 164	8	Ille et Vilaine, France, 1986
*Pseudocercosporella herpotrichoides *var. *acuformis*	C39A	5	1988
*Pseudocercosporella herpotrichoides *var. *acuformis*	PHA 20/3	3	*Secale cereale*, 1991
*Rhizoctonia cerealis*	INRA 161	8	Marne, France, 1981
*Rhizoctonia cerealis*	SAGW 105	5	*Triticum aestivum*, Loxstedt-Dünenfähr, Germany, 1984
*Rhizoctonia cerealis*	SAGW 107	5	Oldenburg, Germany, 1985
*Septoria nodorum*	7n/II/2	5	*Triticum aestivum*, leaf, Göttingen, 1993

**Table 2:** Limits of detection for *F. culmorum *and *F. graminearum *in duplex PCR with melting curve analysis.

**DNA used as template**		**Species detected**	
*F. graminearum*	*F. culmorum*	*F. graminearum*	*F. culmorum*

5 pg	5 pg	**+**	**+**
100 pg	100 pg	**-**	**+**
100 pg	10 pg	**+**	**+**
10 pg	100 pg	**-**	**+**
5 pg to 5 ng	-	**+**	**-**
-	5 pg to 5 ng	**-**	**+**

Positive detection (+) was defined as the presence of a peak in the melting profile which fulfills these conditions:

(i) its melting point identified by thermocycler software lies within 91.0 ± 1.0°C for *F. culmorum *and 86.5 ± 1.0°C for *F. graminearum*

(ii) absolute peak area amounts to at least 100 densitometry units

(iii) the relative peak area in relation to the total area under the melting curve amounts to at least 10%.

Identical results were obtained in the presence of an excess (10 ng) of wheat DNA.

**Table 3:** Influence of SYBR Green I concentration on the melting temperature of the PCR product specific for *F. graminearum*. 50 pg of Fg3 DNA were used as a template for PCR with the primer pair Fg16N F/R in addition to different SYBR Green I concentrations and 3 mM MgCl_2_. The other parameters were set as described in the Methods.

**SYBR Green I concentration in the amplification mix**	**Melting temperature of the 280 bp PCR product**
0.1×	84.5°C
0.2×	85.0°C
0.3×	85.5°C
0.4×	86.0°C
0.7×	86.5°C

## References

[B1] Parry DW, Jenkinson P, McLeod L (1995). Fusarium ear blight (scab) in small grain cereals – A review. Plant Pathology.

[B2] Pieters MN, Freijer J, Baars BJ, Fiolet DCM, van Klaveren J, Slob W (2002). Risk assessment of deoxynivalenol in food: Concentration limits, exposure and effects. Mycotoxins and Food Safety.

[B3] Peraica M, Radic B, Lucic A, Pavlovic M (1999). Toxic effects of mycotoxins in humans. Bulletin of the World Health Organization.

[B4] D'Mello JPF, Placinta CM, Macdonald AMC (1999). Fusarium mycotoxins: a review of global implications for animal health, welfare and productivity. Animal Feed Science and Technology.

[B5] Anonymous (1999). Verordnung über Höchstmengen an Mykotoxinen in Lebensmitteln (Mykotoxin-Höchstmengenverordnung – MHmV). Bundesgesetzblatt.

[B6] Anonymous (2004). Verordnung zur Änderung der Mykotoxin-Höchstmengenverordnung und der Diätverordnung. Bundesgesetzblatt.

[B7] Pirgozliev SR, Edwards SG, Hare MC, Jenkinson P (2003). Strategies for the control of Fusarium head blight in cereals. European Journal of Plant Pathology.

[B8] Waalwijk C, Kastelein P, de Vries I, Kerenyi Z, van der Lee T, Hesselink T, Kohl J, Kema G (2003). Major changes in *Fusarium *spp. in wheat in the Netherlands. European Journal of Plant Pathology.

[B9] Xu XM (2003). Effects of environmental conditions on the development of Fusarium ear blight. European Journal of Plant Pathology.

[B10] Snijders CHA (1990). Systemic fungal growth of *Fusarium culmorum *in stems of winter wheat. Journal of Phytopathology – Phytopathologische Zeitschrift.

[B11] Bottalico A, Perrone G (2002). Toxigenic *Fusarium *species and mycotoxins associated with head blight in small-grain cereals in Europe. European Journal of Plant Pathology.

[B12] Nicholson P, Chandler E, Draeger RC, Gosman NE, Simpson DR, Thomsett M, Wilson AH (2003). Molecular tools to study epidemiology and toxicology of Fusarium head blight of cereals. European Journal of Plant Pathology.

[B13] Schilling AG, Moller EM, Geiger HH (1996). Polymerase chain reaction-based assays for species-specific detection of *Fusarium culmorum*, *F. graminearum*, and *F. avenaceum*. Phytopathology.

[B14] Nicholson P, Simpson DR, Weston G, Rezanoor HN, Lees AK, Parry DW, Joyce D (1998). Detection and quantification of *Fusarium culmorum *and *Fusarium graminearum *in cereals using PCR assays. Physiological and Molecular Plant Pathology.

[B15] Edwards SG, O'Callaghan J, Dobson ADW (2002). PCR-based detection and quantification of mycotoxigenic fungi. Mycological Research.

[B16] Reischer GH, Lemmens M, Farnleitner A, Adler A, Mach RL (2004). Quantification of *Fusarium graminearum *in infected wheat by species specific real-time PCR applying a TaqMan Probe. Journal of Microbiological Methods.

[B17] Reischer GH, Lemmens M, Farnleitner A, Adler A, Mach RL (2004). Erratum to "Quantification of *Fusarium graminearum *in infected wheat by species specific real-time PCR applying a TaqMan probe" [J. Microbiol. Methods 59 (2004) 141–146]. Journal of Microbiological Methods.

[B18] Schena L, Nigro F, Ippolito A, Gallitelli D (2004). Real-time quantitative PCR: a new technology to detect and study phytopathogenic and antagonistic fungi. European Journal of Plant Pathology.

[B19] McCartney HA, Foster SJ, Fraaije BA, Ward E (2003). Molecular diagnostics for fungal plant pathogens. Pest Management Science.

[B20] Giglio S, Monis PT, Saint CP (2003). Demonstration of preferential binding of SYBR Green I to specific DNA fragments in real-time multiplex PCR. Nucleic Acids Research.

[B21] Downloads Petr Karlovsky group. http://www.gwdg.de/~instphyt/karlovsky/downloads/DuplexPCRFusarium.xls.

[B22] Murray MG, Thompson WF (1980). Rapid isolation of high molecular-weight plant DNA. Nucleic Acids Research.

[B23] Stewart CN, Via LE (1993). A rapid CTAB DNA isolation technique useful for rapid fingerprinting and other PCR applications. Biotechniques.

